# Identification and evaluation of reference genes for qRT-PCR studies in *Lentinula edodes*

**DOI:** 10.1371/journal.pone.0190226

**Published:** 2018-01-02

**Authors:** Quanju Xiang, Jin Li, Peng Qin, Maolan He, Xiumei Yu, Ke Zhao, Xiaoping Zhang, Menggen Ma, Qiang Chen, Xiaoqiong Chen, Xianfu Zeng, Yunfu Gu

**Affiliations:** 1 College of Resource, Sichuan Agricultural University, Chengdu, Sichuan, P.R. China; 2 Rice Research Institute of Sichuan Agricultural University, Chengdu, Sichuan, P.R. China; 3 Horticulture Research Institute, Chengdu Academy of Agriculture and Forestry Science, Chengdu, Sichuan, P.R. China; Institute for Sustainable Plant Protection, C.N.R., ITALY

## Abstract

*Lentinula edodes* (shiitake mushroom) is a common edible mushroom with a number of potential therapeutic and nutritional applications. It contains various medically important molecules, such as polysaccharides, terpenoids, sterols, and lipids, were contained in this mushroom. Quantitative real-time polymerase chain reaction (qRT-PCR) is a powerful tool to analyze the mechanisms underlying the biosynthetic pathways of these substances. qRT-PCR is used for accurate analyses of transcript levels owing to its rapidity, sensitivity, and reliability. However, its accuracy and reliability for the quantification of transcripts rely on the expression stability of the reference genes used for data normalization. To ensure the reliability of gene expression analyses using qRT-PCR in *L*. *edodes* molecular biology research, it is necessary to systematically evaluate reference genes. In the current study, ten potential reference genes were selected from *L*. *edodes* genomic data and their expression levels were measured by qRT-PCR using various samples. The expression stability of each candidate gene was analyzed by three commonly used software packages: geNorm, NormFinder, and BestKeeper. Base on the results, *Rpl4* was the most stable reference gene across all experimental conditions, and *Atu* was the most stable gene among strains. *18S* was found to be the best reference gene for different development stages, and *Rpl4* was the most stably expressed gene under various nutrient conditions. The present work will contribute to qRT-PCR studies in *L*. *edodes*.

## Introduction

The basidiomycete *Lentinula edodes* (Berkeley) Pegler (*Lentinus edodes*), an edible mushroom (shiitake), is the most economically important cultivated mushroom in East Asia and the second most popular in the world [1]. *L*. *edodes* is valued for its nutritional and medicinal properties, and has wide culinary and industrial applications [2]. Various compounds with therapeutic properties have been isolated from the mycelium and fruiting body in the last few decades, among which the polysaccharide lentinan has been studied intensively. Lentinan is a type of β-glucan, with high immuno-potentiating, anti metastasic [3,4], antitumor [5,6], antibacterial, antifungal, and antidiabetic activities [7,8]. *L*. *edodes* extracts have caries preventive activity, reduce oral biofilm formation, and have anti-gingivitis effects, accordingly they are extremely useful for the maintenance of oral health [9]. The biochemical pathways involved in the synthesis of high-value lentinan have not been thoroughly characterized. In addition, *L*. *edodes* is a heterothallic homo-basidiomycete, that can be easily cultivated in laboratories and therefore is used for studies of mushroom genetics and physiology [10]. Therefore, insight into the molecular biological mechanisms of *L*. *edodes* is of great value as it provides necessary information about target metabolite synthesis and accumulation.

Analyses of the expression patterns of genes can provide valuable information regarding gene function. Various techniques, such as northern blotting, quantitative real-time reverse transcription polymerase chain reaction (qRT-PCR), gene chips [11], and RNA-seq [12], are applied to study gene expression. Owing to its high sensitivity, repeatability, and specificity, qRT-PCR represents one of the most-widely used technologies for expression analyses. However, the accuracy of qRT-PCR results depends on various factors, including the initial RNA quality and quantity, primer specificity, reverse transcription efficiencies, amplification efficiency, PCR conditions, and transcript normalization. Normalization of qRT-PCR data with suitable internal reference gene(s) is one method to obtain accurate and reliable results of qRT-PCR [13]. House-keeping genes are generally stably expressed under various conditions, and accordingly are considered effective reference genes. However, previous studies have reported that the expression stability of commonly used housekeeping genes is inappropriate for data normalization under a range of experimental conditions. For example, actin is unstably expressed in potato or cucumber in response to salinity stress [14–16]. Therefore, before using reference genes for data normalization, it is necessary to select and evaluate their expression stability under given experimental treatments.

Studies of the selection and evaluation of reference genes have been performed in various species [15,17–24]. However, studies of reference genes for qRT-PCR normalization in *L*. *edodes* are lacking, thereby limiting our ability to optimize the selection of reference genes across growth stages or culture conditions. In addition, the release of the *L*. *edodes* genome sequence has provided an opportunity to scan and validate reference genes for qRT-PCR normalization [25]. In the current study, ten genes were selected as candidate reference genes for the normalization of qRT-PCR data in *L*. *edodes*. These reference genes were evaluated in various strains, developmental stages, and nutrient conditions. Three statistical algorithms, geNorm, NormFinder, and BestKeeper, were used to identify and evaluate the expression stability of candidate reference genes. These results provide useful guidelines to select reference gene(s) for gene expression studies in *L*. *edodes* and also provide a basis for further studies of qRT-PCR data normalization in other fungi.

## Materials and methods

### Sampling and culture conditions

Xin 808, a common artificially cultivated strain in China and other Asia countries, was used. The fungus was grown on potato dextrose agar plates for 7 days at 25°C. Three mycelial discs from the actively growing peripheral region were inoculated into 50 mL of the given medium.

To collect samples reared under different nutrient conditions, two different media were used, i.e., carbon and nitrogen media. The carbon media consisted of the following components (g L^-1^): peptone, 2.5; KH_2_PO_4_, 0.1; MgSO_4_, 0.1; vitamin B1, 0.01; and three different carbon sources (glucose, sucrose or starch), 2.5. The nitrogen media consisted of the following components (g L^-1^): glucose, 2.5; KH_2_PO_4_, 0.1; MgSO_4_, 0.1; vitamin B1, 0.01; and two different nitrogen sources (yeast extract or glycine), 2.5. The cultures were statically incubated at 25°C in the dark and collected after 30 days.

Four strains were included: three cultivated strains (Xin808, Xiang240 and Susheng69) and one wild variety (LSM-1). The vegetative mycelia of these strains were cultured in liquid potato dextrose medium. The culture conditions were the same as those described above.

The primordia and fruiting bodies of Xin808 were collected from PiXian (Chengdu, Sichuan Province, China). The contents of cultivation bag were as follows: sawdust 81%, wheat bran 18%, gypsum 1%, KH_2_PO_4_ 0.1%, magnesium sulfate 0.05%, and water to 55%-60%. All samples were immediately frozen in liquid nitrogen and stored at -80°C for RNA extraction.

### Total RNA extraction and cDNA synthesis

The frozen samples were ground to a fine powder in liquid nitrogen, and 100 mg of the material was used for RNA isolation. Total RNA was extracted using the TRIzol reagent (Invitrogen, Carlsbad, CA, USA) according to the manufacturer’s instructions. The purity and quantity of samples were measured using a Nano spectrophotometer (ND-1000 Thermo Scientific, Waltham, MA, USA), and integrity was checked by agarose gel electrophoresis. First-strand cDNA was synthesized from 2.0 μg of total RNA using a reverse transcription kit (TaKaRa, Shiga, Japan). The synthesized cDNA was diluted 10 times with nuclease-free water and stored at -20°C.

### Reference gene selection and primer design

Ten housekeeping genes were selected as candidate reference genes in this study. Three genes that have been used as reference genes in *L*. *edodes* were selected: *18S* (18S ribosomal protein, KM015456), *28S* (28S ribosomal protein, DQ071718), and *Pma* (plasma membrane proton ATPase, AF146054) [26,27]. In addition, α-Tubulin (*Atu*), β-tubulin (*Btu*), adenine phosphoribosyl transferase (*Apt*), F-actin, Ribosomal protein L2 (*Rpl2*), Ribosomal protein L4 (*Rpl4*), and tryptophan synthase-β (*Tsb*) were also selected on the basis of previous studies of other species [18].

Since sequence information for *L*. *edodes* is limited, publicly available gene sequences from *Gymnopus luxurians* (http://genome.jgi.doe.gov/Gymlu1/Gymlu1.home.html), the most closely related species to *L*. *edodes*, were used to obtain gene sequences for *L*. *edodes* [25]. The specific reference gene sequences of *L*. *edodes* were obtained from the genome database of *L*. *edodes* (http://legdb.chenlianfu.com/), using *G*. *luxurians* homologus DNA sequences (CDS) as a queries.

Gene-specific primers were designed using Primer-Blast (https://www.ncbi.nlm.nih.gov/tools/primer-blast/). The criteria were as follows: amplified products ranging from 140 to 200 bp, Tm around 60°C, and GC% content around 55%. The primers were synthesized by Shenggong Co., LTD. (http://www.sangon.com/) and are presented in Table 1.

**Table 1:** Candidate genes, primers, and different parameters derived from the qRT-PCR analysis.

Gene	Gene symbol	Primer sequences (5’-3’)	Amplicon Size (bp)	Tm (^o^C)	E (%)	Regression coefficient (R^2^)
18S ribosomal protein	*18S*	For GCGCTACACTGACAGAGCCA	178	82.4±0.22	110	0.981
		Rev GCGGTGTGTACAAAGGGCAG				
28S ribosomal protein	*28S*	For TGGCTCTAAGGGTTGGGTGC	129	82.0±0.19	93	0.996
		Rev CCCGAAGAGCAGCCAAAGTC				
plasma membrane proton ATPase	*Pma*	For GGTGCGTTCTGTGTACCCCA	157	81.2±0.25	97	0.994
		Rev TCGCGAACTTGATCCAGTCGA				
α-Tubulin	*Atu*	For ATCTGTAACGAACCCCCAGC	169	83.3±0.35	96	0.946
		Rev CACCGACGTACCAGTGAACA				
β-tubulin	*Btu*	For CAGTTCACGGCCATGTTCA	152	82.5±0.16	91	0.965
		Rev CGACGGTGGCATCCTGGTA				
adenine phosphoribosyl transferase	*Apt*	For AGGAAGTGAAATGGCCGAGG	161	82.0±0.08	101	0.963
		Rev TGATGCGAAGAGCCCATTCA				
ribosomal protein L4	*Rpl4*	For AATCGTAGACACCGTCAGCG	160	82.5±0.00	110	0.938
		Rev TGACGAAACGGCCAAGATGA				
ribosomal protein L2	*Rpl2*	For AATCGATCCCTCGGGAAAGC	181	84.5±0.11	95	0.974
		Rev CAGGCTTCCTTGTCCTCGTT				
tryptophan synthase-β	*Tsb*	For CTGGTGTTGGACCTGAGCAT	183	82.6±0.15	99	0.986
		Rev CCTTAGGAAGCGTCTTGGCA				
UDP-glucose pyrophosphorylase	*UGPase*	For GACGGCCAAGGGGTTATTCA	165	83.0±0.00	110	0.979
		Rev TTGACCGTGGCTCAAAGAGT				
phosphoglucose isomerase	*PGI*	For TCCATCAGGGCACCAAACTC	141	82.0±0.00	109	0.981
		Rev CGGTCTTACCGAAGGCCAAT				
phosphoglucomutase	*PGM*	For CCCATGCCGACGAATACAGA	176	84.5±0.00	96	0.985
		Rev GGTGTGAGCGTAGGTCAAGT				

### Quantitative real-time PCR

Real-time amplification reactions were performed in 96 well plates using SYBR Green detection chemistry and run in triplicate on 96-wells plates with the iCycler iQ5 thermo-cycler (Bio-Rad, USA). Reactions were prepared in a total volume of 20 μL containing: 1μL of 10 fold diluted template, 0.5μL of each amplification primer (1μM), 5μL SsoFast™ EvaGreen® Supermix (Bio-Rad, USA), and nuclease-free water to a final volume of 20 μL. Non-template controls were also included for each primer pair. Reactions were carried under the following conditions: 95°C for 2 min, followed by 40 cycles of 95°C for 15 s,63°C for 20 s and 72°C for 26 s. A melting curve analysis was generated by heating the amplicon from 60 to 90°C to confirm that a single product was generated by each reaction. Thermal cycling, fluorescence data collection, and data analysis were performed using the iCycler iQ5 Thermo-cycler (Bio-Rad, Hercules, CA, USA) detection system according to the manufacturer’s instructions.

Standard RT-PCR was performed for all primer pairs and amplification products of the expected size for each gene were confirmed by electrophoresis on a 2% agarose gel. The primer amplification efficiency was determined from a standard curve generated by serial dilutions of cDNA (10-fold each) for each gene in triplicate. Correlation coefficients (*R*^*2*^ values) and amplification efficiencies (E) for each primer pair were calculated from the slope of the regression line by plotting mean Cq values against the log cDNA dilution factor in Microsoft Excel using the equation E (%) = (10^(1/-slope^−1) *100.

### Data analysis

To facilitate the analysis of the expression stability of candidate reference genes, samples that differ with respect to developmental stage (Development), strain (Strain), and nutrient source (Nutrient), and all test samples (All). Three publicly available software tools, i.e., geNorm (version 3.5) [28], NormFinder (version 0.953) [29], and BestKeeper [30], were used to analyze expression variation among the ten candidate reference genes. Data were exported to Excel and converted to appropriate input files according to the software requirements.

### Validation of reference genes analysis

To confirm the effectiveness of the selected reference genes for data normalization, expression levels of UDP-glucose pyrophosphorylase (*UGPase*), phosphoglucose isomerase (*PGI*) and phosphoglucomutase (*PGM*), which play important roles in the biosynthesis of polysaccharides, were examined in different developmental stages. According to the comprehensive ranking for samples obtained at various developmental stages, *Btu* and *18S* were the most two stable genes and were selected as reference genes for the expression analysis. Additionally, the least stable reference gene, F-actin, was used to analyze the influence of the selection of an inappropriate reference gene.

The qRT-PCR amplification conditions were the same as those described above. The relative expression levels of the three genes were standardized according to the formula Y (%) = 10 –^(ΔCt/3)^ × 100, where ΔCt is the difference between the cycle threshold value of the target gene and the reference gene [31,32].

## Results

### Selection and identification of candidate reference genes

A total of 10 candidate reference genes, including three genes (*18S*, *28S*, and *Pma*) that have already been used as reference genes and seven new genes: *Atu*, *Btu*, *Apt*, F-actin, *Rpl2*, *Rpl4*, and *Tsb* were chosen in this study.

**Fig 1:**
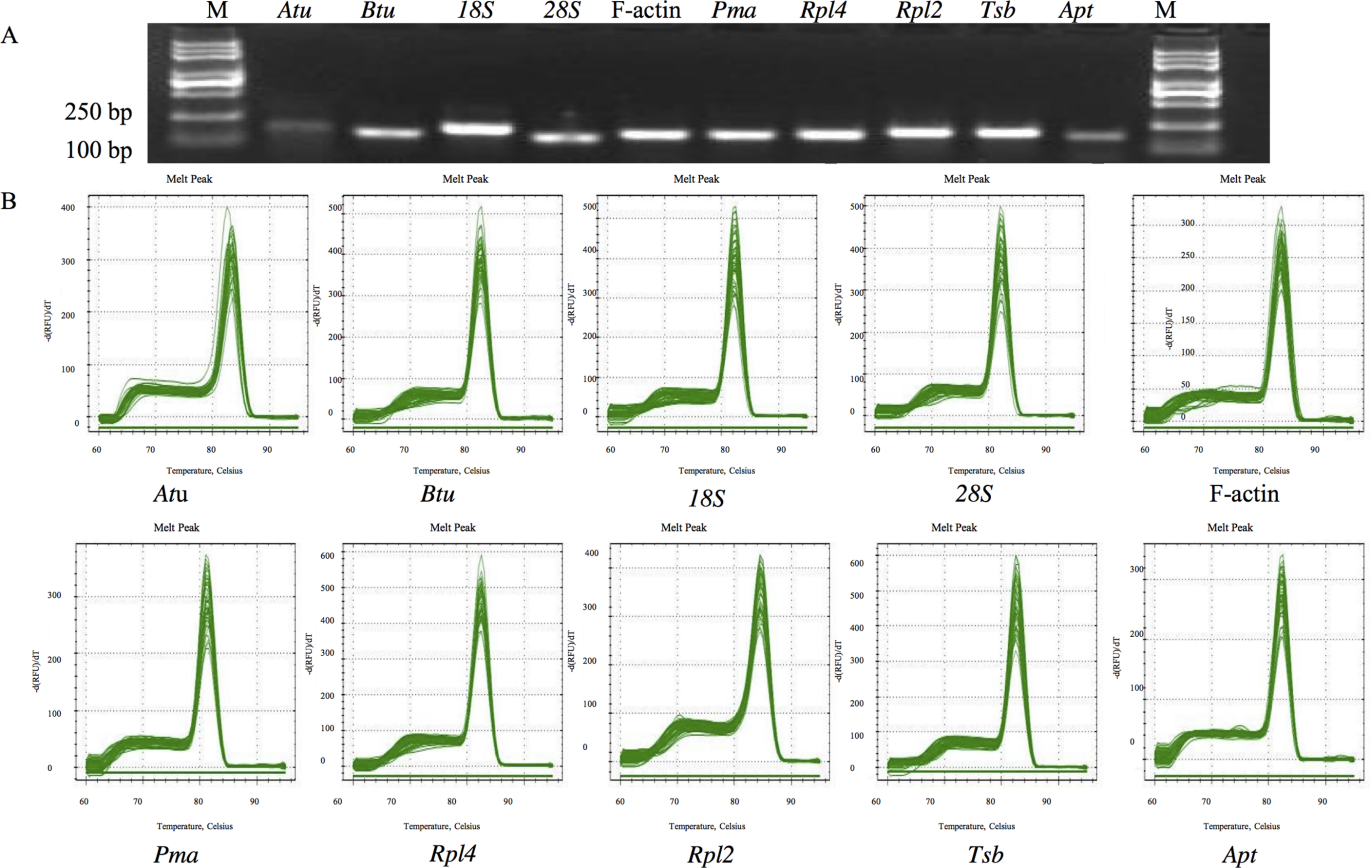
The amplicon length and specificity of candidate reference genes. A: Amplified fragments of candidate reference genes shown by agarose gel electrophoresis with ethidium bromide staining. B: Melting curves generated by qRT-PCR. For each sub-graph, temperature is displayed in the x axis, the derivative reporter signal is displayed in the y axis.

To determine the specificity of the primers used in this study, agarose gel electrophoresis and a melting curve analysis were performed following the qRT-PCR experiment. All primer pairs amplified a single PCR product of the expected size (Fig 1), without any nonspecific amplicons or primer dimers. The specificity of the amplicon was also confirmed by melting curves generated by qRT-PCR (Fig 1) and a sequencing analysis. A standard curve was generated using 10-fold serial dilutions of cDNA to calculate the gene-specific PCR efficiency. The amplification efficiencies for the 10 genes varied from 89% (F-actin) to 110% (*Rpl4* and *18S*), and the correlation coefficients ranged from 0.938 for Rpl4 to 0.996 for 28S. The T_m_ ranged from 81.2°C for Pma to 84.5°C for Rpl2 (Table 1).

### Expression profiles of reference genes

**Fig 2:**
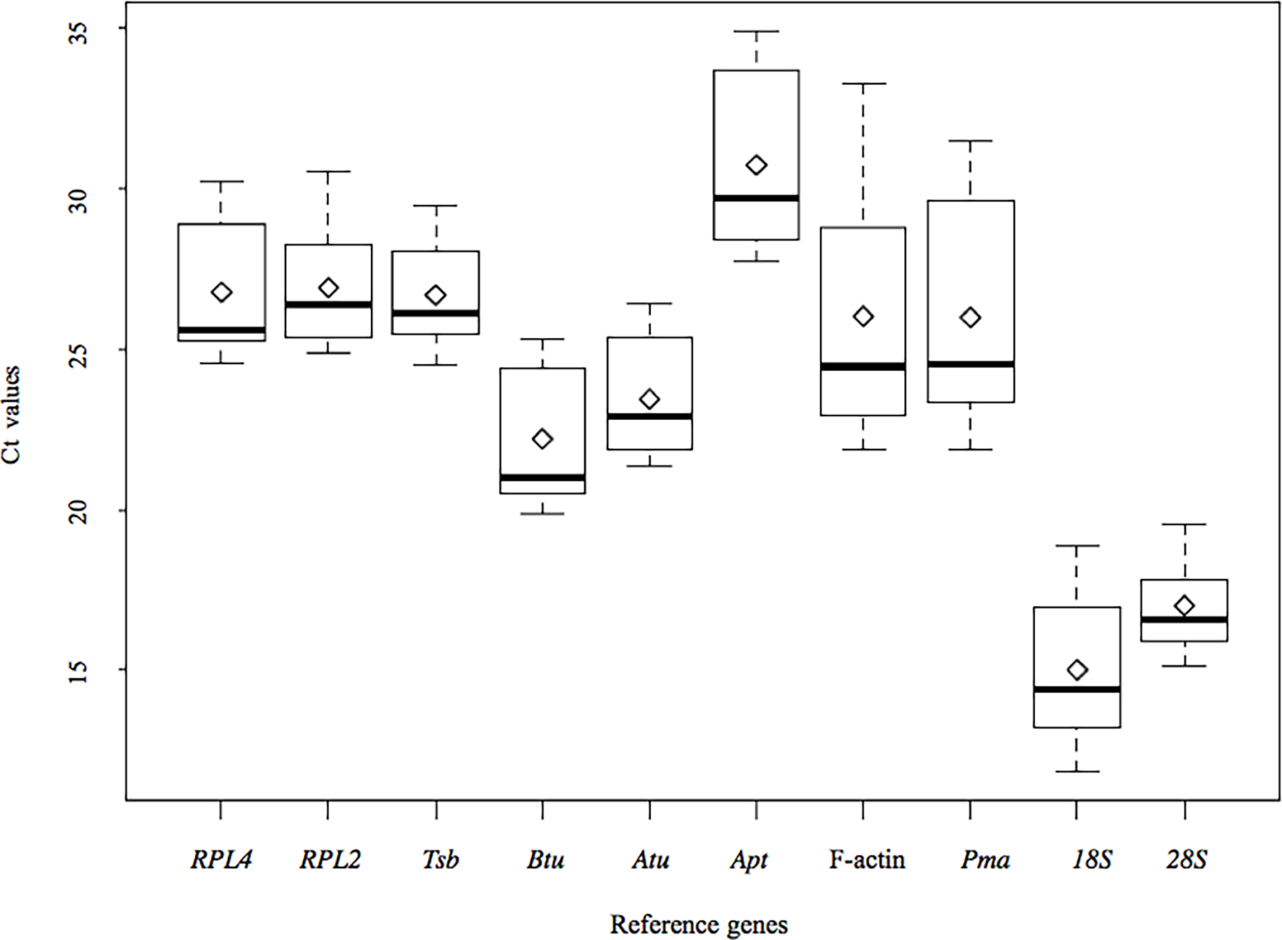
Distribution overview of expression profiles of different reference genes. Boxplot representation of raw Ct values obtained from amplification curves. The box indicates the 25^th^ and 75^th^ percentiles. Whiskers represent the maximum and minimum values, the thin line within the box marks the median and mean (rhombus).

The expression stability of each candidate reference gene was evaluated by measuring Ct values for each individual sample pool. The expression profiles of these reference genes across all samples are shown in Fig 2. The Ct values for the ten reference genes ranged from 11.86 (*18S* in a maltose fermentation sample) to 34.89 (*Apt* in the primordium of *L*. *edodes*), showing a wide range of variation. The average Ct value for each reference gene was ~ 24.11, with remarkable variation (15.00 for *18S* to 30.73 for *Apt*). Greater than 84% of Ct values were between 16 and 30. The lowest gene expression variation among all the studied samples was observed for *28S* (15.14–19.55), whereas the expression level of *Apt* was the most variable (21.88–33.25). The remaining eight reference genes exhibited intermediate variation (Fig 2). The wide range of Ct values for the ten reference genes indicated that none of the reference genes had a constant expression pattern in different samples. Therefore, in order to select a proper and stable reference gene to normalize gene expression data in *L*. *edodes*, it is necessary to evaluate the expression stabilities of these candidate reference genes under particular conditions.

### Reference gene stability analysis

Three common algorithms, i.e., geNorm, NormFinder, and BestKeeper, were used to evaluate the ten reference genes. GeNorm (v3.5) yields M values by the stepwise exclusion of unstable genes, followed by the recalculation of M [28]. Stably expressed genes have M values of less than 1.5, and the most stable gene has the lowest M value [28]. The expression stability was further analyzed using Microsoft Excel-based NormFinder, by comparing the variation within and between user-defined sample groups [29]. NormFinder provides a stability value (SV) for each candidate gene, which is a direct measure of the expression variation. A lower SV value corresponds to higher gene expression [29]. BestKeeper finds the most stably expressed genes by estimating the coefficient of correlation (*r*) and BestKeeper Index (geometric mean) [30]. The most stable reference gene shows the lowest value *r* value and standard deviation (CV±SD). Candidate genes that show a SD value of less than 1 are considered to be reliable references [19, 33–35]. The samples used in this study were divided into three subgroups to evaluate the expression stability of candidate genes using these three softwares. The first subgroup consisted of four divisions based on strains (Strain), the second subgroup consisted of three divisions based on the different developmental stages of Xin808 (Development), and the third subgroup consisted of five divisions based on the nutrient source of Xin808 (Nutrient). In addition, all samples were analyzed (All).

### Reference genes expression among strains

The ranking of the ten reference genes from different strains obtained with the three algorithms indicated that *Pma* was the least stably expressed gene (S1 Fig). For most of the reference genes, the rankings obtained using NormFinder and geNorm were quite similar. However, greater variations was observed using BestKeeper, consistent with previous reports [15,17,36]. Each reference gene was evaluated with respect to stability using the three tests (Table 2), and *Atu* was the most stable gene, followed by *Rpl4*.

**Table 2:** Overall ranking of the candidate reference genes for the four sample groups after geNorm, NormFinder, and BestKeeper analysis.

Candidate reference genes											
		*Rpl4*	*Btu*	*Rpl2*	*Tsb*	*Atu*	*Apt*	*18s*	*28s*	*Pma*	F-actin
Strain	geNorm	1	3	5	6	2	7	4	9	10	8
	Normfinder	2	3	6	7	1	5	4	9	10	8
	BestKeeper	7	5	3	2	6	9	4	1	10	8
	Total score	10	11	14	15	9	21	12	19	30	24
	Overall rank*	**2**	**3**	**5**	**6**	**1**	**8**	**4**	**7**	**10**	**9**
Development	geNorm	7	3	4	6	8	9	1	2	5	10
	Normfinder	7	3	3	6	8	9	2	1	5	10
	BestKeeper	3	4	6	9	1	2	5	8	7	10
	Total score	17	10	13	21	17	20	8	11	17	30
	Overall rank	**5**	**2**	**4**	**9**	**5**	**8**	**1**	**3**	**5**	**10**
Nutrient	geNorm	1	3	5	4	2	7	6	10	8	9
	Normfinder	1	3	6	5	2	7	4	10	8	9
	BestKeeper	5	7	2	3	4	8	6	1	9	10
	Total score	7	13	13	12	8	22	16	21	25	28
	Overall rank	**1**	**4**	**4**	**3**	**2**	**8**	**6**	**7**	**9**	**10**
All	geNorm	1	2	3	4	5	6	7	8	9	10
	Normfinder	1	2	4	5	6	3	7	8	9	10
	BestKeeper	5	6	3	2	4	8	7	1	9	10
	Total score	7	10	10	11	15	17	21	17	27	30
	Overall rank	**1**	**2**	**2**	**4**	**5**	**7**	**8**	**6**	**9**	**10**

*The overall rank is expressed in boldface.

### Reference gene expression among various developmental stages

The stability of the ten candidate reference genes among developmental stages was evaluated for all three samples. F-actin exhibited wide variation in Ct values (Fig 2) and was ranked the least stable gene according to all three algorithms (S2 Fig). *18S*, was the most stable gene according to geNorm and was ranked second and fifth by NormFinder and BestKeeper, respectively. However, *Pma*, which was the least stable reference gene among strains, was identified as the fourth most stable gene among developmental stages. In all cases, *18S* was the gene identified as the most suitable reference gene for the normalization of qRT-PCR data for samples obtained at different developmental stages. *Btu* and *28S* are the next best gens on the basis of their overall rankings.

### Reference gene expression for various nutrient conditions

The ranking of the reference genes for different nutrient conditions varied according to the three algorithms (S3 Fig). *28S* was the least stable gene according to both geNorm and NormFinder, but was the most stable gene according to BestKeeper. *Rpl4* was the best gene according to geNorm and NormFinder and was ranked fifth by BestKeeper. The three least stable genes were the same as those identified on the basis of expression profiles, i.e., F-actin, *Pma*, and *Apt* (Fig 2). *Rpl4* was identified as the most stable gene, followed by *Atu* (Table 2). It may be noticed that the ranking of the ten candidate reference genes was quite different among sample groups.

### Reference gene expression in all samples

**Fig 3:**
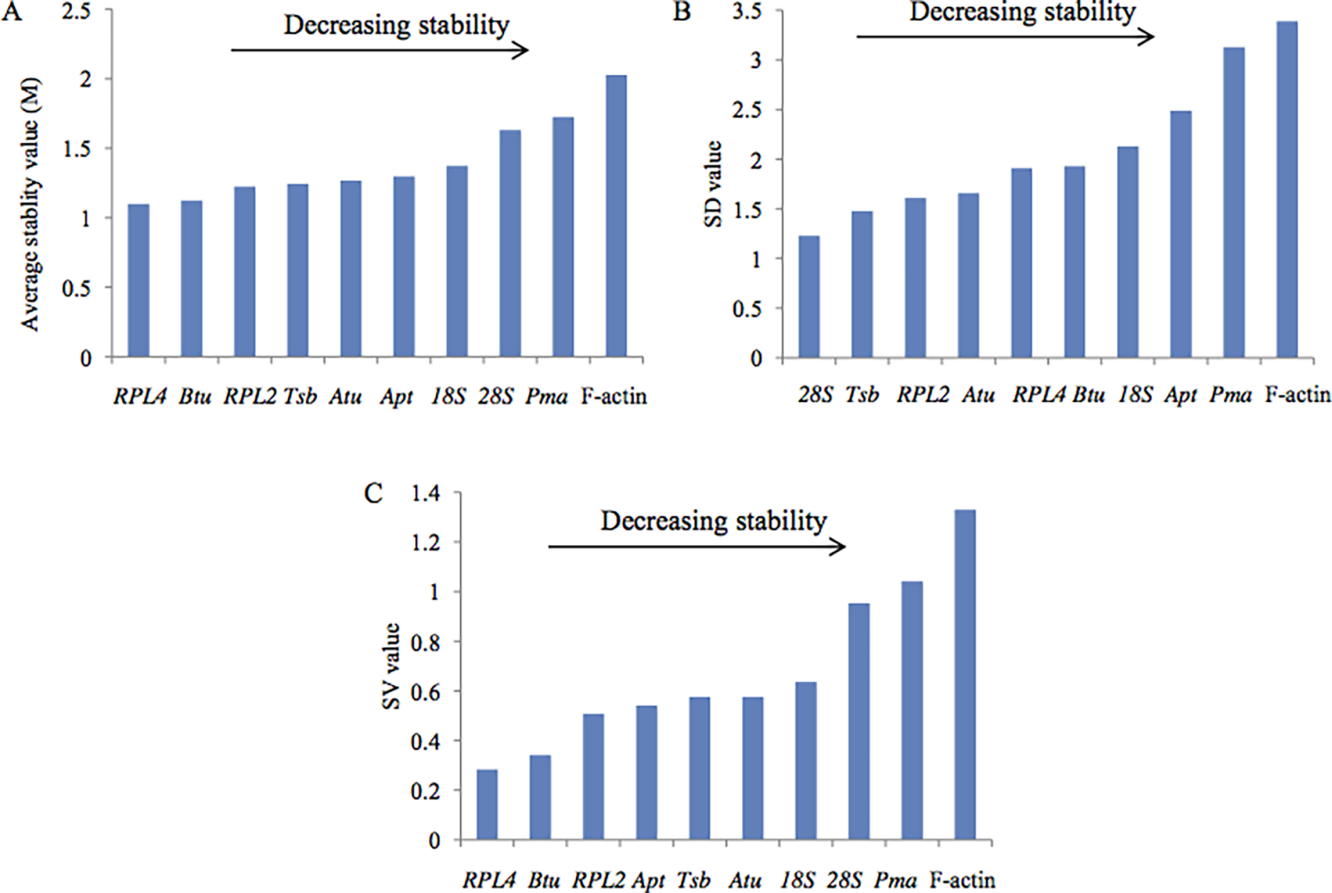
Ranking of the candidate reference genes for all samples according to geNorm (A), BestKeeper (B), and NormFinder.

To identify reference genes suitable for all the samples, the stability of the ten candidate reference genes was evaluated for all 11 samples and the results are presented in Fig 3. *Pma* and F-actin were still identified as the two least stable genes by all three algorithms. *Rpl4* and *Btu*, which were ranked as the top two genes by both geNorm and NorFinder, was identified as fifth and sixth most stable genes by BestKeeper. Using the stability ranking of each reference gene in all three tests, *Rpl4* was identified as the most stable gene, followed by *Btu* and *Rpl2* (Table 2).

### Determination of the optimal number of reference genes for normalization

**Fig 4:**
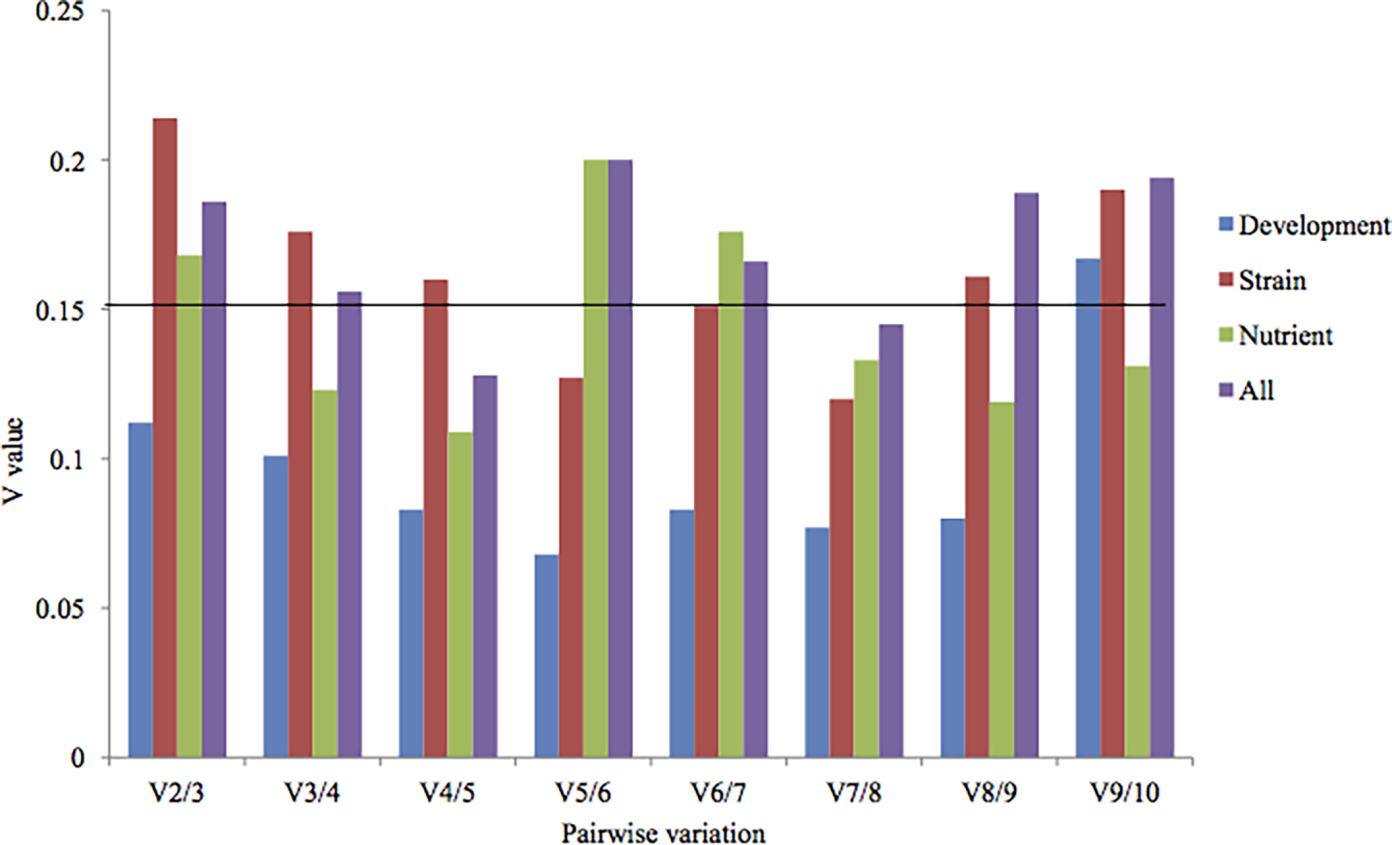
Pairwise variation calculated by geNorm between Vn and Vn + 1to determine the minimum number of reference genes required for accurate normalization in four different samples. The cut off value is 0.150, below which the inclusion of an additional reference gene is not required.

Some reports have suggested that the use of more reference genes may lead to more stable results [28,37]. To determine the optimal number of reference genes in each experimental condition, pairwise variation (V) was calculated using geNorm by applying a cutoff value of 0.150 [28]. A pairwise variation analysis showed that the optimal number of reference genes may be different for distinct samples. In the pairwise variation analysis for different developmental stage samples, the V2/3 value was less than 0.150, showing that only two reference genes is suffieient for normalizing gene expression in these samples (Fig 4). The pairwise variation among various nutrient sample conditions was V2/3 0.168, which is above the cutoff of 0.150, and the addition of next reference gene decrease this value to 0.130, implying that proper normalization required at least three reference genes. As a consequence, for nutrient samples, *Rpl4*, *Atu* and *Tsb* can be used as reference genes to normalize gene expression data. Similarly, the V5/6 value for various strains samples was 0.127, indicating that five reference genes are required to normalized gene expression strains. Accordingly, *Atu*, *Rpl4*, *Btu*, *18S*, and *Rpl2* can be used for data normalization in various strains. For all samples combined, the pairwise variation at V3/4 was 0.156, and with an additional gene it decreases to 0.128, indicating that at least four reference genes are needed for normalization. Therefore, *Rpl4*, *Btu*, *Rpl2*, and *Tsb* and can be used to normalize gene expression data for these samples.

## Validation of reference genes

**Fig 5:**
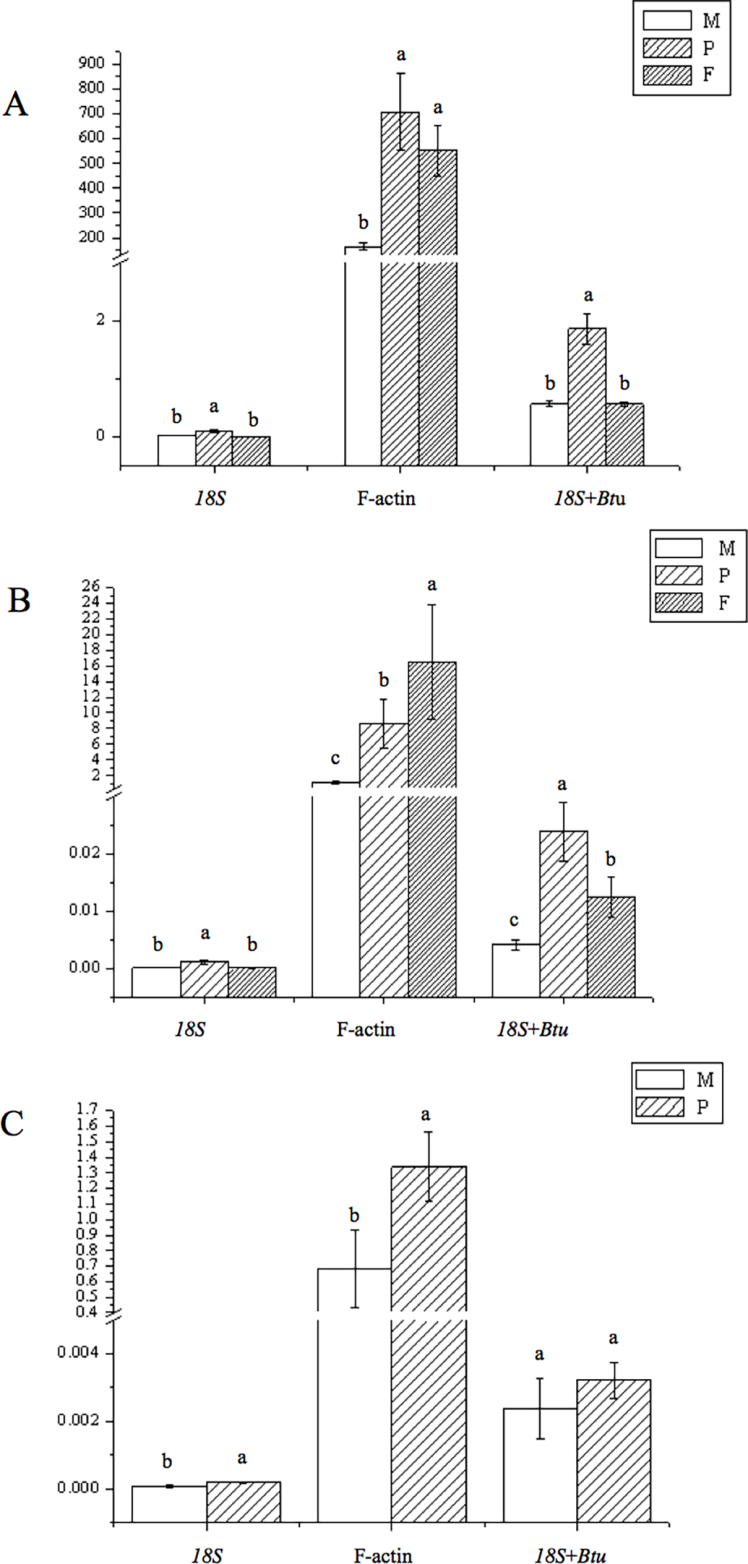
**Relative expression levels of *UGPase* (A), *PGI* (B) *and PGM*(C) among various developmental stages of *L*. *edodes*.** M: mycelium; P: primordial; F: fruiting bodies. The most stable genes recommended for Development sample (*18S* and *Btu*) and the least stable gene F-actin were used for normalization. For *18S* and *Btu* geometric mean was calculated and used for normalization of expression. Error bars show standard deviation calculated from three biological replicates. The relative expression levels of the three genes were indicated as percentage to the expression of candidate reference genes. Different letters above histograms indicate statistical difference highlighted using ANOVA (p-value < 0.05).

To validate the selection of candidate reference genes, the relative expression levels of three key genes (*UGPase*, *PGI* and *PGM*) involved in the biosynthesis of polysaccharides during various growth stages, including in the mycelium, primordium and fruiting body, were monitored. The results revealed that the three genes were differentially or specifically expressed (Fig 5). The relative expression levels of *UGPase* and *PGI*were higher in the fruiting-body than at the other two stages. No transcript of *PGM* was detected in the fruiting-body stage. Relative expression levels of *PGM* obtained by normalizing against “*18S*+*Btu*” or *18S* alone indicated similar trends. However, normalization against F-actin led to the opposite conclusion, indicating that expression was higher in the fruiting-body stage.

## Discussion

Among various technologies, qRT-PCR is considered the most appropriated method for gene expression analyses. However, several quality control measures can improve the reliability of results, including the validation of suitable reference genes for data normalization. In order to screen for suitable genes for data normalization in *L*. *edodes*, expression stabilities of ten house-keeping genes were analyzed using three software packages: geNorm, NormFinder, and Bestkeeper. The three statistical algorithms have been used to select and validate reference genes for qRT-PCR data normalization across a variety of tissues, organs, developmental stages, stress conditions in various plant species like tomato [20], pepper [21], bamboo [22], and fungi like (e.g.,*Ganoderma lucidum*) [18], and some specific conditions, e.g., in the rice blast fungus *Magnaporthe oryzae* [38] or plum under different postharvest treatments [24]. To our knowledge, the selection and validation of reference genes in *L*. *edodes* have not been evaluated.

In this study, expression levels as well as the stability of ten candidate reference genes were measured in *L*. *edodes* using three different algorithms. The candidate reference genes showed a relatively wide range of expression profiles under different conditions (Fig 2), confirming that no single gene had a constant expression profile. The three algorithm yielded quiet different results with respect to the ranking of the ten candidate reference genes, indicating the importance of using more than one software type to achieve the best results. *Rpl4* was identified as the most stable gene for various nutrient conditions and all samples by NormFinder and geNorm, and was also recommended as the best reference gene for qRT-PCR data normalization in the entire sample in *G*. *lucidum* [18]. The NormFinder and geNorm methods ranked *Atu* as the first and second most stable genes among strains, respectively.*18S* was found to be the most stable gene according to geNorm, and NormFinder ranked 18S second, but Bestkeeper ranked it fifth. F-actin, a classic reference gene frequently used as an internal control for RT-PCR normalization in plants and animals, was identified as the least stable gene under nutrient, developmental variation and in all samples used in this study. Other than for the set of all samples, we can see that the top three reference genes were almost the same for all groups when determined by geNorm and NormFinder, and not by BestKeeper. This variation in the results may be explained by difference in the algorithms implemented by the three software packages.

It has been reported that the transcription levels of Apt and *Pma* remains constant throughout all stages of fruiting body development (mycelium, primordium, young fruiting body and mature fruiting body) in *L*. *edodes* [26], indicating that these two genes are stably expressed and can be used as an internal controls to study gene expression profiles for samples obtained at different developmental stage. However, *Apt* gene was ranked eighth for Development, Strain and Nutrient samples in this study (Table 2). This difference may be due to differences among strains used in the two studies. *Pma*, which has been used as an internal control to study fruiting body development in *L*. *edodes* [39], was ranked fifth by geNorm and NormFinder, and seventh by BestKeeper for different developmental samples (Table 2), indicating that this gene is not suitable for developmental sample data normalization. These results emphasize the importance of validating reference genes for each experimental design.

In recent years, intensive research has focused on polysaccharide biosynthesis owing to its function as a host defense potentiator and thereby its important antivirus, antitumor, and immune function regulation roles among other functions. The polysaccharide content varied among developmental stages [40]. Hence, we analyzed the expression of three key genes involved in the polysaccharide biosynthetic pathway at different developmental stages. UGPase catalyzes the reversible production of uridine diphosphate glucose (UDPG) and pyrophosphate (PPi) from Glc-1-P and UTP, which is a key regulatory step for carbohydrate metabolism in many species because it is an universal precursor involved in biosynthesis of many carbohydrates, including sucrose, glucan, cellulose, hemicellulose, the carbohydrate moiety of glycolipids, and glycoproteins [41]. Higher expression levels of *UGPase* were detected in primordium stages than in other stages. PGM is involved in conversion of glucose-6-phosphate to glucose-1-phosphate, and there is a linear relationship between its activity and the production of EPS [42]. No transcript of *PGM* was observed in the fruiting-body stage, and its relative expression level in the primordium was higher than that in the fruiting-body stage Since PGI catalyzes the reversible isomerization of D-glucopyranose-6-phosphate and D-fructofuranose-6-phosphate, and the reaction that generates D-fructose-6-phosphate (F6P), it plays dual roles in EPS [43]. The transcript level of PGI was higher in the fruiting-body than in other developmental stages. The expression patterns of these three genes showed highly similar trends when the most stable reference genes *18S* and *Btu* were used, either singly or in combination. However, a different conclusion, i.e., that the expression of *PGI* in the fruiting-body stage was higher than that in the other two stages, was obtained when the most unstable gene, F-actin, was used as a reference gene. These results further implied that the use of an unstable reference gene could lead to the misinterpretation of gene expression results.

In conclusion, the present work provides basic background information regarding reference gene selection for qRT-PCR studies in *L*. *edodes*. The study contributes to will make a contribution for further genomics and transcriptomics studies of this valuable medicinal fungus.

## Supporting information

S1 Fig Ranking of the candidate reference genes for different strain samples according to geNorm (A), BestKeeper (B), and NormFinder (C).
(TIFF)

S2 Fig Ranking of the candidate reference genes for different development stage samples according to geNorm (A), BestKeeper (B), and NormFinder (C).
(TIFF)

S3 Fig Ranking of the candidate reference genes for different nutrientt samples according to geNorm (A), BestKeeper (B), and NormFinder(C).
(TIFF)
