# Supplementary figures and images for: Identification and evaluation of reference genes for qRT-PCR studies in Lentinula edodes
Source: PLoS One. 2018 Jan 2;13(1):e0190226. doi: 10.1371/journal.pone.0190226 (PMC5749753; doi:10.1371/journal.pone.0190226)

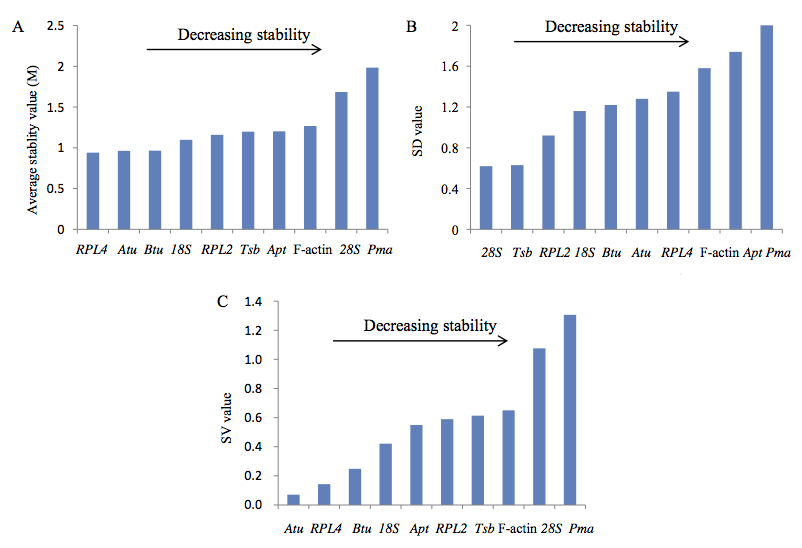

Supplement: S1 Fig — Ranking of the candidate reference genes for different strain samples according to geNorm (A), BestKeeper (B), and NormFinder (C). (TIFF) [file pone.0190226.s001.tiff]

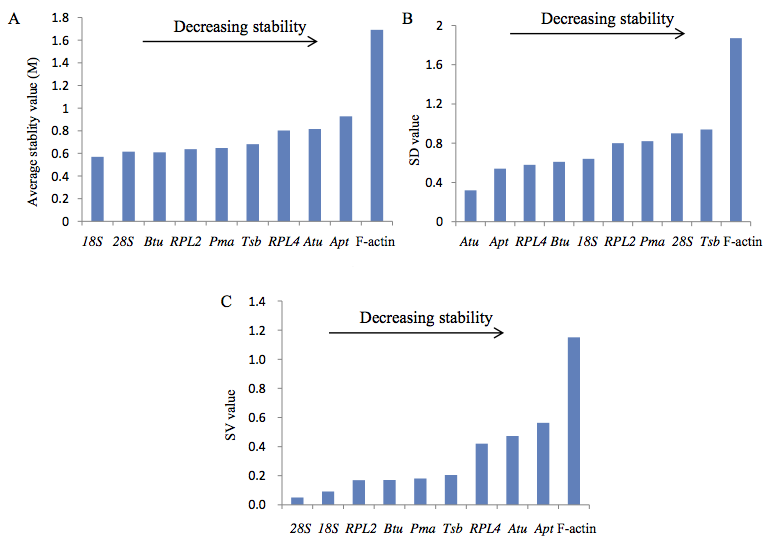

Supplement: S2 Fig — Ranking of the candidate reference genes for different development stage samples according to geNorm (A), BestKeeper (B), and NormFinder (C). (TIFF) [file pone.0190226.s002.tiff]

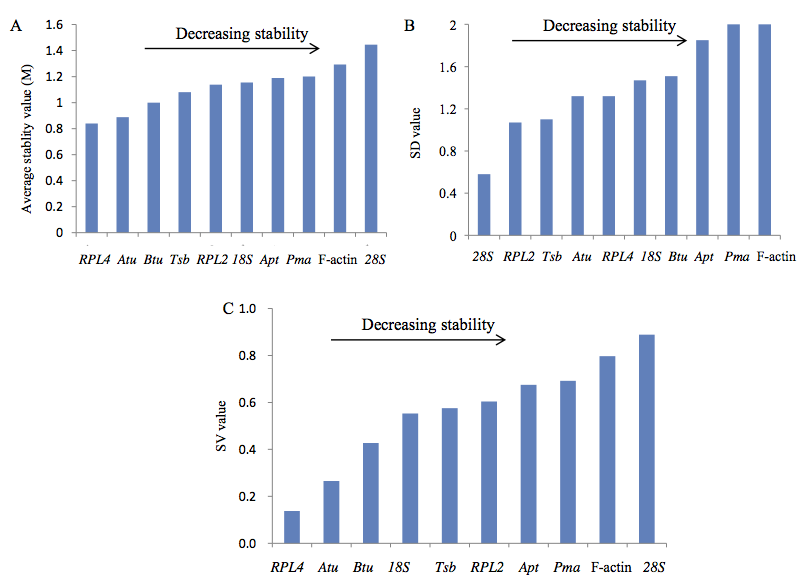

Supplement: S3 Fig — Ranking of the candidate reference genes for different nutrientt samples according to geNorm (A), BestKeeper (B), and NormFinder(C). (TIFF) [file pone.0190226.s003.tiff]
